# Dairy Cattle, a Potential Reservoir of Human Campylobacteriosis: Epidemiological and Molecular Characterization of *Campylobacter jejuni* From Cattle Farms

**DOI:** 10.3389/fmicb.2018.03136

**Published:** 2018-12-18

**Authors:** Jae-Uk An, Hungwui Ho, Jonghyun Kim, Woo-Hyun Kim, Junhyung Kim, Soomin Lee, Seung-Hyun Mun, Jae-Ho Guk, Sahyun Hong, Seongbeom Cho

**Affiliations:** ^1^BK21 PLUS Program for Creative Veterinary Science Research, Research Institute for Veterinary Science and College of Veterinary Medicine, Seoul National University, Seoul, South Korea; ^2^Veterinary Research Institute, Ipoh, Malaysia; ^3^Division of Bacterial Disease Research, Center for Infectious Diseases Research, Korea National Institute of Health, Centers for Disease Control and Prevention, Cheongju-si, South Korea; ^4^Division of Bacterial Disease, Center for Laboratory Control of Infectious Diseases, Centers for Disease Control and Prevention, Cheongju-si, South Korea

**Keywords:** *Campylobacter jejuni*, risk factor analysis, prevalence, dairy cattle farms, virulence genes, molecular subtyping

## Abstract

*Campylobacter jejuni* is a major foodborne pathogen that is increasingly found worldwide and that is transmitted to humans through meat or dairy products. A detailed understanding of the prevalence and characteristics of *C. jejuni* in dairy cattle farms, which are likely to become sources of contamination, is imperative and is currently lacking. In this study, a total of 295 dairy cattle farm samples from 15 farms (24 visits) in Korea were collected. *C. jejuni* prevalence at the farm level was 60% (9/15) and at the animal level was 23.8% (68/266). Using the multivariable generalized estimating equation (GEE) model based on farm-environmental factors, we estimated that a high density of cattle and average environmental temperature (7 days prior to sampling) below 24°C affects the presence and survival of *C. jejuni* in the farm environment. Cattle isolates, together with *C. jejuni* from other sources (chicken and human), were genetically characterized based on analysis of 10 virulence and survival genes. A total of 19 virulence profile types were identified, with type 01 carrying eight genes (all except *hcp* and *virB11*) being the most prevalent. The prevalence of *virB11* and *hcp* was significantly higher in isolates from cattle than in those from other sources (*p* < 0.05). Multilocus sequence typing (MLST) of *C. jejuni* isolates from three different sources mainly clustered in the CC-21 and CC-48. Within the CC-21 and CC-48 clusters, cattle isolates shared an indistinguishable pattern with human isolates according to pulsed-field gel electrophoresis (PFGE) and *flaA*-restriction fragment length polymorphism (RFLP) typing. This suggests that CC-21 and CC-48 *C. jejuni* from dairy cattle are genetically related to clinical campylobacteriosis isolates. In conclusion, the farm environment influences the presence and survival of *C. jejuni*, which may play an important role in cycles of cattle re-infection, and dairy cattle represent potential reservoirs of human campylobacteriosis. Thus, environmental management practices could be implemented on cattle farms to reduce the shedding of *C. jejuni* from cattle, subsequently reducing the potential risk of the spread of cattle-derived *C. jejuni* to humans through the food chain.

## Introduction

*Campylobacter* is a major bacterial pathogen causing human gastroenteritis worldwide (Acheson and Allos, 2001). *Campylobacter* infection of humans is generally accompanied by abdominal pain and convulsions and rarely with a neurological syndrome called Guillain–Barré syndrome (Kimoto et al., 2006). Of the causes of campylobacteriosis, *Campylobacter jejuni* accounts for 90% of human infections in most parts of the world (Gillespie et al., 2002). A 2017 U.S. FoodNet surveillance study reported 9,421 (38.48%) cases of *Campylobacter* infection among 24,484 bacterial foodborne infections (Marder et al., 2018). In South Korea, campylobacteriosis outbreaks have been continuously occurring (Ministry of Food, and Drug Safety, 2018).

Although poultry has been reported to be one of the leading sources of human campylobacteriosis, ruminants are responsible for the second highest number of human *C. jejuni* infections (Stanley and Jones, 2003; Mughini Gras et al., 2012). According to U.S. Centers for Disease Control (CDC) data (Centers for Disease Control and Prevention, 2017), 209 *Campylobacter* outbreaks were reported in the United States from 2010 to 2015, among which 72 were reportedly derived from dairy sources and 33 from poultry sources. At the farm level, cattle infected with *Campylobacter* may shed bacteria, increasing the risk of infection of other animals or humans through contamination of the environment (Quinn, 1998). Therefore, if the contaminated farm environment is not managed properly, *Campylobacter* shed in the feces of dairy cattle could be easily transmitted to humans through dairy products such as unpasteurized milk (Bianchini et al., 2014). Furthermore, it has recently been reported that multistress-tolerant *C. jejuni* strains in raw milk can survive high-temperature, short-time (HTST) pasteurization processes (Oh et al., 2018). Therefore, understanding the prevalence of *C. jejuni* on dairy cattle farms is essential for disease control and prevention.

*Campylobacter jejuni* strains can recombine and readily take up DNA from the environment, resulting in high genetic diversity (Young et al., 2007). Because of this genetic diversity, *C. jejuni* may express a wide variety of toxin- and pathogenicity-associated genes depending on the source. Besides the direct expression of toxin genes, such as *cdt*, *C. jejuni* may have factors that determine pathogenicity, such as those that affect motility, chemotaxis, adhesion, invasion, and the production of toxins that affect the host (Bolton, 2015). Structurally, *Campylobacter* strains feature secretion systems that play a role in virulence, such as type III (T3SS), type IV (T4SS), and type VI (T6SS) secretion systems – encoded by *flhB*, *virB11*, and *hcp*, respectively – which transport protein toxins from the bacterial cytoplasm into the host or transport extracellular factors (Koolman et al., 2015). In particular, *hcp* (hemolysin co-regulated protein), which encodes the T6SS, was recently found to play roles in virulence by influencing cell adhesion, cytotoxicity toward red blood cells, and colonization (Harrison et al., 2014). Another study has shown that patients infected with *hcp*-positive *C. jejuni* develop bloody diarrhea more frequently than patients infected with *hcp*-negative *C. jejuni* (Harrison et al., 2014). As campylobacteriosis is mainly caused by foodborne transmission, it is vulnerable to exposure to heat and oxidative stress (Bolton, 2015). As a microaerophilic microbe, *Campylobacter* is susceptible to oxidative stress in the food-production chain, and thus studies of genes such as *csrA*, *katA*, *sodB*, *perR*, and *htrA*, which are involved in stress resistance, are needed to better understand the potential pathogenicity to hosts (Bolton, 2015; Kim et al., 2015; Koolman et al., 2015).

The subtyping of isolates from distinct sources generates epidemiological linkage information that may be useful for assessing future infection risk, controlling disease, and identifying *Campylobacter* infection sources (Han et al., 2007). Among subtyping methods, multilocus sequence typing (MLST) is a molecular epidemiology tool for tracing the origin of *C. jejuni* strains from a weak clonal population (Dingle et al., 2001). Pulsed-field gel electrophoresis (PFGE), which is highly discriminatory, is a widely recognized standard technique used by PulseNet to subtype *Campylobacter*, and *flaA*-restriction fragment length polymorphism (RFLP) typing is regarded as an easy, rapid, and commonly used genotyping method for discriminating among *Campylobacter* isolates (Nielsen et al., 2000). Combining these molecular epidemiological methods allows for an in-depth analysis of the relationships among *C. jejuni* isolates.

The objectives of this study were to (a) determine the prevalence of *C. jejuni* among dairy cattle farms in Korea and identify farm-environmental factors that might affect the pathogen’s farm-level prevalence; (b) compare the characteristics of *C. jejuni* isolates from cattle, chicken, and human sources based on putative virulence and survival-related genes; and (c) investigate the genetic relatedness of isolates by using molecular subtyping methods to identify the potential risks of *C. jejuni* on dairy cattle farms.

## Materials and Methods

### Cattle Farm Samples and Data Collection

A total of 295 samples were collected over 24 visits from 15 dairy cattle farms located in Gyeonggi-do, Korea between August 2012 and September 2013 (Supplementary Table S1), including 266 samples of cattle feces and 29 bedding samples. During each visit, 4–36 fecal samples and 1–2 bedding samples were collected. The number of cattle on the studied farms ranged from 30 to 250 (median: 60, mean: 86). For first sampling in 2012, The number of samples in each farm was determined by a stratified random sampling method and classified according to the herd size of each farm. The second sampling was conducted in 2013 for the farms selected as high risk for *C. jejuni* through the first sampling in 2012.

In addition, farm-environmental factors associated with *C. jejuni* occurrence on dairy cattle farms were investigated. The density of the lactating herd was categorized as normal or high based on the guidelines of the Livestock Industry Act [Enforcement Date 22 Sept., 2017; Act No. 14654, 21 Mar., 2017, Partial Amendment] (Ministry of Government Legislation, 2017). The sawdust hygiene level was estimated based on the methods of a previous study (Reneau et al., 2005) and categorized as low, moderate, and high. We also investigated whether the farm experienced stamping-out due to a foot-and-mouth disease (FMD) outbreak in 2010–2011 (2 years prior). The main feed used for adult cattle was categorized as total mixed ration (TMR) or hay. Environmental temperature data were gathered from the Korea Meteorogical Administration database based on the average temperature of the 7 days prior to the sampling date in the area in which the farm was located; temperature was classified into two groups: above and below 24°C (Korea Meteorological Administration, 2018).

### Isolation and Identification of *C. jejuni*

One gram of each sample was homogenized with 9 mL of Bolton broth containing 5% hemolyzed horse blood and a Bolton broth selective supplement (Oxoid, United Kingdom). The samples were incubated at 42°C for 48 h microaerobically. On the following day, one loop of broth was streaked onto modified charcoal cefoperazone deoxycholate agar (mCCDA) containing CCDA selective supplement (Oxoid), and then the samples were incubated at 42°C for 48 h microaerobically. Next, two to six suspected colonies from the mCCDA plates were picked, subcultured in blood agar, and incubated microaerobically at 42°C for 48 h. To identify *C. jejuni* isolates, multiplex PCR targeting the 16S rRNA gene and *cj0414* and singleplex PCR targeting *hipO* were performed using the DNA templates, according to methods described previously (Yamazaki-Matsune et al., 2007; Supplementary Table S2).

### Bacterial Strains

Total of 68 *C. jejuni* strains were isolated from 266 fecal samples in 15 dairy cattle farms and 3 *C. jejuni* strains were isolated from 32 bedding samples. For further analysis, 58 strains (57 from individual cattle feces, and 1 from bedding sample) were selected based on the individual prevalence on each farm. In addition, two isolates from slaughterhouses, collected in 2014 were used for analysis.

For comparison, a total of 103 *C. jejuni* strains from different sources were analyzed together, including 52 strains selected from our previous studies on chickens (5 from farms, 24 from slaughterhouses, and 23 from retail meats) and 51 strains from human clinical cases through the nationwide surveillance system of Korea Centers for Disease Control & Prevention (KCDC). American Type Culture Collection (ATCC) strains 33560 and 700819 (NCTC 11168) were used as reference strains.

### Statistical Analysis of Farm-Environmental Factors Associated With *C. jejuni* Occurrence

Generalized estimating equations (GEEs) (SPSS, IBM) were used to analyze the farm-environmental factors affecting the prevalence of *C. jejuni* on dairy farms by considering the characteristics of each farm and the clustering effects of repeated visits. A negative binomial model with log link and offset (the number of samples per visit) was used to correct for the sampling distribution. All significant variables (*p* < 0.05) in the univariable model were included as candidate variables in the initial multivariable GEE model. The backward stepwise elimination method was used to remove the variable with the highest *p*-value, until all variables remaining in the final model were significant (*p* < 0.05).

### DNA Extraction

DNA templates were prepared using a simple boiling method. Briefly, isolates from blood agar were suspended in 200 μL of distilled water and boiled for 10 min, placed on ice for 3 min, and centrifuged at 13,000 × *g* for 3 min. The supernatant was used to identify the presence of *C. jejuni*, detect the presence of virulence and survival-related genes, and perform *flaA*-RFLP and MLST. If the *flaA* gene was not amplified clearly, DNA template extracted using the HiGene^TM^ Genomic DNA Prep Kit (BioFact, Korea). *C. jejuni* ATCC 33560 and nuclease-free distilled water were used as positive and negative controls, respectively, for the PCR assay.

### Virulence and Survival-Related Genes in *C. jejuni*

PCR was performed using a PCR mixture (final volume, 20 μL) that contained 1× EmeraldAmp GT PCR Master Mix (Takara, Japan) and 0.25 mM each of forward and reverse primers targeting gene encoding the following: three virulence factors belonging to the type III, IV, and VI secretion systems in *Campylobacter* (*flhB*, *virB11*, and *hcp*, respectively); an outer membrane fibronectin-binding protein associated with adhesion (*cadF*); two cell invasion-related proteins, phospholipase (*pldA*) and ABC transporter ATP-binding protein (*iamA*); cytolethal distending toxin B (*cdtB)*, the representative toxin identified in *Campylobacter*; and the survival-related factors responsible for biofilm formation (*csrA*), oxidative-stress resistance (*perR*), and heat tolerance (*htrA*) (González-Hein et al., 2013; Kim et al., 2015; Supplementary Table S2). Here, the primer for *perR* was designed using Primer-BLAST software based on the consensus sequence generated by aligning four *perR* genes (accession numbers: AL111168, EF569030, EF569029, and EF569028) using CLUSTALW in the MegAlign program (DNASTAR, United States). The primer sequences and PCR conditions are summarized in Supplementary Table S2, and the PCR products were electrophoresed and visualized on 1% agarose gels.

### Subtyping Methods

Multilocus sequence typing targeted the internal fragments of seven *Campylobacter* housekeeping genes (aspartase, *aspA*; glutamine synthetase, *glnA*; citrate synthase, *gltA*; serine hydroxy methyltransferase, *glyA*; phosphoglucomutase, *pgm*; transketolase, *tkt*; and ATP synthase alpha subunit, *uncA*) with primer sets from a PubMLST protocol. Each nucleotide sequence amplified by PCR was sequenced with an ABI PRISM 3730XL DNA analyzer (Applied Biosystems, United States). Nucleotide sequence data were submitted to PubMLST, and sequence type (ST) and clonal complex (CC) profiles were determined.

Pulsed-field gel electrophoresis procedures were based on the CDC’s PulseNet protocol, with slight modifications. *C. jejuni* cell suspensions in 0.85% NaCl were adjusted to 4.0 on the McFarland scale using a DensiCHEK Plus instrument (bioMérieux, France). Three slices from the lysed and washed plug were cut and incubated at 25°C for 2-h digestion with 20 U of *Sma*I (Takara). Electrophoresed gels loaded with sample isolates were viewed under a Gel Doc XR (Bio-Rad, United States). *Salmonella* Braenderup ATCC BAA664 was used as the size ladder marker.

To perform *fla*A-RFLP typing, *flaA* was amplified using primers adopted from previous studies (Nachamkin et al., 1996; El-Adawy et al., 2013; Supplementary Table S2). The 25-μL PCR assay mixture was prepared using 1× PCR buffer, 2 mM MgCl_2_, 200 μM deoxynucleoside triphosphate (dNTP), 0.2 μM each of forward and reverse primers, AmpliTaq Gold^®^ (1.5 U), 1 μL of DNA template, and distilled water. The PCR products were electrophoresed to check for the presence of the 1.7-kb *flaA* gene; if the *flaA* gene was not amplified, PCR was performed again using a wobble reverse primer. After band confirmation, 8 μL of each PCR product was digested with 5 U of *Dde*I and 1 × CutSmart^TM^ Buffer (New England BioLabs, United States) in a final volume of 20 μL and then electrophoresed in a 2% agarose gel at 100 V for 2 h. Gels were viewed under a Gel Doc XR. The size-standard marker used with the digested products was a 1 kb Plus DNA Ladder (BioFact, Korea).

### Data Analysis of Molecular Subtyping

A Chi-square test was used to analyze differences in the prevalence of virulence and survival-related genes between sources. Statistically significant differences showed *p* < 0.05 in two-tailed tests.

Band profiles from PFGE, *flaA-*RFLP typing, and MLST of seven housekeeping gene alleles were analyzed using BioNumerics software version 6.6 (Applied Maths, United States). Dendrograms of PFGE and *flaA-*RFLP typing results were generated based on the Dice similarity coefficient with the unweighted pair group method with arithmetic mean (UPGMA) using a tolerance of 1.5%. Isolates that exhibited ≥90% similarity were assigned to the same genotypic pattern. A UPGMA dendrogram and minimum spanning tree (MST) were generated based on the allelic profile categorical values of each of the seven housekeeping genes.

The Simpson’s diversity (1-D) and Shannon’s diversity (H′) indices of the PFGE, *flaA*-RFLP typing, and composite analysis data were calculated to evaluate discriminatory power, as described previously (Boxrud et al., 2007).

## Results

### Farm-Environmental Factors Associated With *C. jejuni* Prevalence on Dairy Farms

In this study, the farm-level prevalence of *C. jejuni* was 60.0% (9/15), and the animal-level prevalence was 25.6% (68/266). The animal-level prevalence for each farm visit ranged from 0.0 to 80.0% (median: 18.4%, mean: 25.5%). Of the 32 bedding samples collected from each farm, three samples (9.4%) were positive for *C. jejuni*.

Among the six farm-environmental variables, four variables were significantly associated with *C. jejuni* prevalence on dairy farms based on univariable GEE model analysis. Potential risk factors identified in this univariable analysis included low to moderate sawdust hygiene levels [odds ratio (OR): 9.24, 95% confidence interval (CI): 2.23–38.25], environmental temperature below 24°C (OR: 5.82, 95% CI: 1.87–18.07), below 100 cattle on the farm (OR: 8.82, 95% CI: 1.31–59.27), and a high density of cattle on the farm (OR: 4.29, 95% CI: 1.14–16.08), as shown in Table 1. In the final multivariable GEE model, two variables were identified as risk factors for *C. jejuni* prevalence on dairy farms (Table 2): high animal density on the farm (OR: 9.97, 95% CI: 3.43–28.98) and environmental temperature below 24°C (OR: 9.45, 95% CI: 2.21–40.45).

**Table 1:** Farm-environmental variables associated with *C. jejuni* prevalence on dairy cattle farms using univariable generalized estimating equations (GEE) model.

		Univariable estimates		
Farm-environmental variables		Odds ratio (OR)	95% Wald confidence interval	*p*-value
Sawdust hygiene level^b^	Low to moderate	9.24	2.23–38.25	0.002^†^
	High	–	–	–
Environmental temperature (average over the 7 days prior to sampling)	Below 24°C (<24°C)	5.82	1.87 18.07	0.002^†^
	Above 24°C (=24°C)	–	–	–
Farm Size	Small (<100 cattle/farm)	8.82	1.31–59.27	0.025^∗^
	Large (≥100 cattle/farm)	–	–	–
Density in farm	High (<8.3 m^2^/cattle)	4.29	1.14–16.08	0.031^∗^
	Normal (≥8.3 m^2^/cattle)	–	–	–
Experience stamping out due to FMD (2010–2011)	Yes	1.55	0.30–7.97	0.600
	No	–	–	–
Main feed	TMR^a^	0.64	0.08–4.81	0.661
	Hay	–	–	–


*^a^TMR, total mixed ration.*

*^b^Hygiene level: high – very dry, moderate – normal, and low – very wet, dirty, and slippery (Reneau et al., 2005).*

*Statistically significant (^∗^p < 0.05, ^†^p < 0.005).*

**Table 2:** Farm-environmental variables associated with *C. jejuni* prevalence on dairy cattle farm using multivariable generalized estimating equations (GEE) model.

		Multivariable estimates		
Farm-environmental variables		Odds ratio (OR)	95% Wald confidence interval	*p*-value
Density in farm	High (<8.3 m^2^/cattle)	9.97	3.43–28.98	<0.001^†^
	Normal (≥8.3 m^2^/cattle)	–		–
Environmental temperature (average over the 7 days prior to sampling)	Below 24°C (<24°C)	9.45	2.21–40.45	0.002^†^
	Above 24°C (≥24°C)	–		–


*Statistically significant (^†^*p* < 0.005).*

### Virulence- and Survival-Associated Genes in *C. jejuni*

Table 3 summarizes the PCR results obtained for the 10 genes associated with virulence and survival in 163 *C. jejuni* isolates (60 from cattle, 52 from chickens, and 51 from humans). *htrA* was present in all isolates tested, and seven other genes (*flhA*, *cdtB*, *perR*, *csrA*, *cadF*, *pldA*, and *iamA*) were present in most isolates (>90%); by contrast, *virB11* and *hcp* were detected in 13/163 (7.98%) and 71/163 (43.56%) isolates, respectively. The prevalence of *virB11* and *hcp* were significantly higher in isolates from cattle than in isolates from other sources (*p* = 0.012 and *p =* 0.002, respectively; Chi-square test). Five of the 10 *virB11* positive isolates from cattle originated from the same farm. By contrast, the prevalence of *cadF*, *pldA*, and *iamA* were lower among cattle isolates than among human and chicken isolates (*p* < 0.001, *p = 0*.001, and *p =* 0.001, respectively; Chi-square test).

**Table 3:** Comparison of virulence and survival-associated genes in *C. jejuni* isolates from different sources.

		Source^a^		
Functional category	Gene	Cattle (*n* = 60)	Chicken (*n* = 52)	Human (*n* = 51)
T3SS^b^	*flhB*	60 (100.0)	51 (98.1)	51 (100.0)
T4SS^b^	*virB11^∗^*	10 (16.7)	2 (3.8)	1 (2.0)
T6SS^b^	*hcp*^†^	35 (58.3)	22 (42.3)	14 (27.5)
Adhesion	*cadF*^†^	35 (81.4)^c^	52 (100.0)	50 (98.0)
Invasion	*pldA*^†^	33 (76.7)^c^	51 (98.1)	48 (94.1)
	*iamA*^†^	35 (81.4)^c^	51 (98.1)	50 (98.0)
Cytotoxin	*cdtB*	58 (96.7)	52 (100.0)	49 (96.1)
Stress response	*csrA*	58 (96.7)	48 (92.3)	50 (98.0)
	*perR*	59 (98.3)	51 (98.1)	50 (98.0)
	*htrA*	60 (100.0)	52 (100.0)	51 (100.0)


*^a^Figures in parentheses refer to the percentage of isolates positive for each gene.*

*^b^T3SS, type III secretion system; T4SS, type IV secretion system; T6SS: type VI secretion system.*

*^c^Prevalence of cadF, pldA, and iamA from cattle represents the ratio among a total of 43 isolates.*

*Prevalence of virB11 and hcp in C. jejuni from cattle was significantly higher than that in isolates from other sources. In the case of cadF, pldA, and iamA, prevalence in C. jejuni from cattle was significantly lower than that from other sources. Statistically significant (^∗^p < 0.05, ^†^p < 0.005).*

**FIGURE 1:**
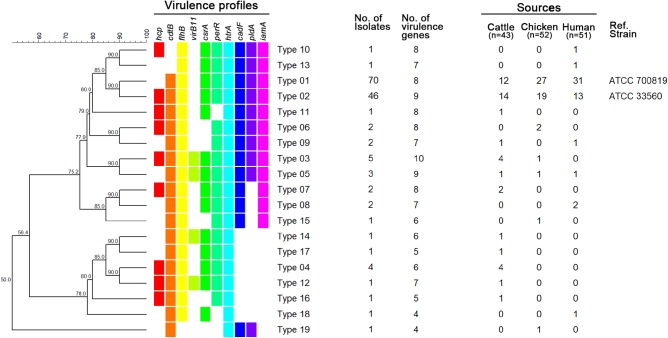
UPGMA dendrogram based on virulence profile types. Virulence-associated genes for each type are indicated in different colors for each gene. Blank, not detected.

A total of 19 virulence profile types were identified, with type 01 containing eight genes (all except *hcp* and *virB11*) and being the most dominant (47.85%; Figure 1). The distributions of virulence profiles differed for each source, with 12, 7, and 8 types present among isolates from cattle, chickens, and humans, respectively. The virulence profile type 01 was the most dominant among *C. jejuni* isolates from chickens (27/52, 51.92%) and humans (31/51, 60.78%), while the virulence profile type 02 was the most common among *C. jejuni* isolated from dairy cattle (14/43, 32.56%). Of the 19 virulence profile types, 14 types were isolated from a single type of source and carried fewer than eight virulence-associated genes. By contrast, at least seven virulence-associated genes were detected in five virulence profile types isolated from multiple sources (Figure 1).

### MLST

**FIGURE 2:**
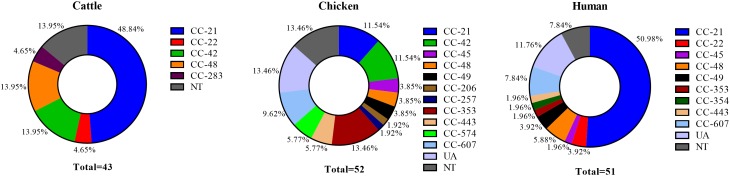
Proportion of MLST clonal complexes in each source. UA, unassigned ST; NT, non-typable ST (including eight different non-typable STs).

Multilocus sequence typing was performed on 146 *C. jejuni* strains (43 from cattle, 52 from chicken, and 51 from human sources), and 48 different STs were identified. Moreover, 14 CCs were assigned, in addition UA (unassigned) and NT (non-typable), and cattle, chicken, and human *C. jejuni* strains were classified into 5, 11, and 9 CCs, respectively, aside from the strains belonging to STs not specified or reported. Four CCs were found to be source-specific (CC-206, 257, 354, and 574). The major CC types were as follows: CC-21 (48.84%), CC-42 (13.95%), and CC-48 (13.95%) among cattle isolates; CC-353 (13.46%), CC-21 (11.54%), and CC-42 (11.54%) among chicken isolates; and CC-21 (50.98%), CC-607 (7.84%), and CC-48 (5.88%) among human clinical isolates (Figure 2). A total of eight NT types were newly identified (two from cattle, five from chicken, and one from human) and were registered in the PubMLST database.

Our results confirmed that CC-21 (36.30%, 53/146) was the major CC, followed by CC-42 (8.22%, 12/146) and CC-48 (7.53%, 11/146), regardless of source. In our MLST cluster analysis, CC-21 and CC-48 contained isolates from all three sources. We found that the proportion of CC-21 in each source was 48.84% (21/43) among cattle and 50.98% (26/52) among humans, which is higher than that in chicken with 11.54% (6/52). The proportion of CC-48, was higher in cattle sources (13.95%, 6/43) than that from human (5.88%, 2/52) and chicken sources (3.85%, 3/52) (Figure 2).

**FIGURE 3:**
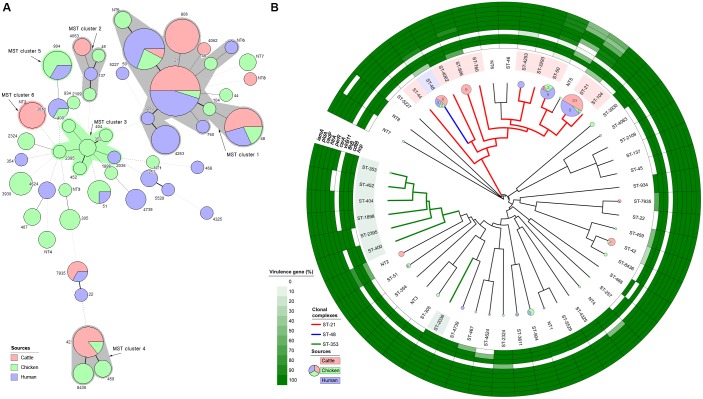
Clonal distribution analysis of *C. jejuni* isolates from all three sources. **(A)** Minimum spanning tree based on MLST data from 146 strains. Each number represents a sequence type, and the size of the node indicates the number of strains belonging to the ST (red: cattle, green: chicken, and purple: human). A bold solid line indicates that there is one allele difference between STs, and a thin solid line indicates that two and three alleles are different according to the length. Four to six allele differences are represented by a wide-interval dotted line, and seven are indicated by a narrow-interval dotted line. Nodes with fewer than two different alleles have been placed in the same cluster. Each cluster contains at least five entries and at least one node. The number shows the sequence type of each node. The color of the shadow represents the major source of the cluster, while a gray shadow indicates no primary source. **(B)** UPGMA dendrogram based on 51 different STs identified through MLST in a total of 146 strains, and detection rate of 10 virulence-associated genes of *C. jejuni* belonging to each ST. Pie chart shows the number of strains belonging to each ST (red: cattle, green: chicken, and purple: human), and the major CC is indicated by a thick, solid, colored line and the ST background color. In addition, the positivity rates of pathogenicity-associated genes in the strains belonging to each ST are shown.

A MST was generated from MLST data using distance-based analysis (Figure 3A). Six clusters were identified, including four major clusters containing two or more different STs and two minor clusters formed by a single ST. MST clusters 1 and 2 contained strains isolated from three different sources. Clusters 3, 4, and 5 consisted of two different sources, and cluster 6 consisted of a single source (dairy cattle). MST cluster 1 consisted of all CC-21 and CC-48 strain, except for two strains (ST-46 and ST-NT5, chicken source).

### PFGE

A total of 163 analyzed isolates were able to be typed using PFGE, and for each isolate, six to nine restriction fragments were produced (Supplementary Figure S1). The PFGE dendrogram contained 66 different genotypes; 27 clusters containing >1 isolate for each genotype were generated, while 69.64% of the genotypes (39/66) were unique (one isolate/genotype). The number of isolates of each allocated genotype ranged from 1 to 19. Six clusters contained isolates originating from different cattle farms, with a maximum of five farm isolates exhibiting indistinguishable PFGE patterns. Among the 27 clusters, two clusters contained isolates from three sources (cattle, chicken, and humans), nine clusters contained isolates from two different sources, and the remaining 16 contained isolates from a single source.

### flaA-RFLP Typing

Among the 163 isolates analyzed using *flaA-*RFLP typing, 159 *C. jejuni* isolates produced 5–10 restriction fragments each (Supplementary Figure S2), and four isolates were untypable. The *flaA-*RFLP typing generated 63 genotypes and 25 clusters, with 38 of the genotypes being unique. The number of isolates per genotype ranged from 1 to 20. Six clusters included isolates from distinct cattle farms, with a maximum of three farms being allocated to the same *flaA-*RFLP type. Among the 25 clusters, two clusters contained isolates from three sources (cattle, chickens, and humans), seven clusters contained isolates from two different sources, and 16 contained isolates from a single source.

### Composite Analysis

The UPGMA dendrogram based on MLST data was used to determine the phylogenetic relationships among STs (Figure 3B). As in the MST analysis (MST cluster 1), CC-21 and CC-48 were genetically similar to each other. Cattle and human strains were predominant in the CC-21 and CC-48 clusters. By contrast, strains from chicken sources were distributed among various STs including CC-353 and CC-607. Analysis of virulence-associated genes revealed that the strains belonging to CC-21 (ST-21, 50, 806, and 4253) and CC-48 (ST-48) were found to exhibit various gene presence/absence patterns (Figure 3B). These two CCs included 14 virulence profile types among a total of 19 different types (Supplementary Figure S3). In ST-21, the prevalence of virulence type 01 was high (35%; 7/20) and that of type 02 was relatively low compared with the proportion among all 146 strains from all sources. By contrast, CC-353 and CC-607 were mainly isolated from chickens and humans, and only four of the 19 virulence profile types were identified, among which type 02 predominated.

**FIGURE 4:**
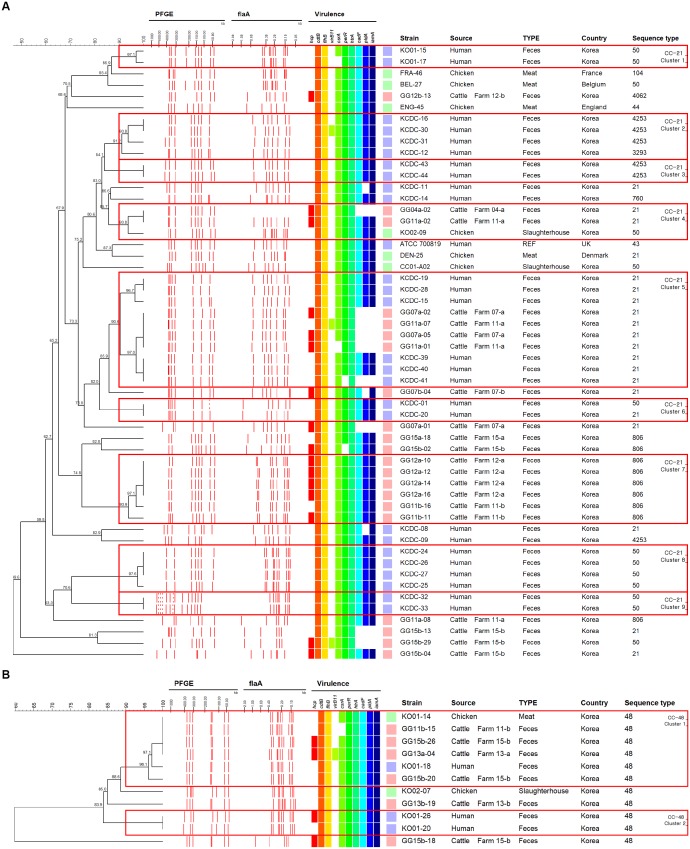
UPGMA dendrogram based on PFGE and *flaA*-RFLP band pattern composite analysis for strains belonging to CC-21 and CC-48. **(A)** Strains belonging to CC-21. **(B)** Strains belonging to CC-48. Dendrograms were generated based on 2% optimization and 1.5% tolerance based on unweighted pair group method with arithmetic mean. Boxes indicate clusters created based on 90% similarity. The letter next to the farm number indicates the visit number (a – first visit and b – second visit).

In CC-21, cattle isolates shared indistinguishable patterns with human isolates based on a composite analysis of PFGE and *flaA*-RFLP typing band patterns (Figure 4A, cluster 5). By contrast, in CC-48, *C. jejuni* isolates from cattle sources were grouped into a single cluster *C. jejuni* isolate human and chicken (Figure 4B, cluster 1). Among dairy cattle isolates clustered with human isolates belonging to both CC-21 cluster 5 and CC-48 cluster 1 (Figures 4A,B), the prevalence of the *hcp* and *virB11* genes were 62.5% (5/8) and 25.0% (2/8), respectively, which were higher than the overall prevalence among all *C. jejuni* strains from cattle (58.3 and 16.7%, respectively).

### Discriminatory Index

The Simpson’s diversity (1-D) and Shannon’s diversity (H′) indices showed different results for each source. The molecular subtyping methods with the highest Simpson’s indices for each source type were as follows: cattle: *flaA*-RFLP typing (0.933), chicken: MLST (0.976), humans: PFGE (0.947), and overall: PFGE (0.967) (Supplementary Table S3). In the case of isolates from domestic chickens only, *flaA*-RFLP typing showed the highest discriminatory power (data not shown).

## Discussion

*Campylobacter jejuni* is the most common causative microbe for bacterial foodborne infections according to a survey by the U.S. FoodNet surveillance system (Marder et al., 2018). In addition, 209 *Campylobacter* outbreaks were reported in the United States from 2010 to 2015; dairy sources were identified as the most common source for these infections (Centers for Disease Control and Prevention, 2017). According to the epidemiologic studies for *Campylobacter* outbreaks, the main cause for outbreak was contaminated raw milk (Davis et al., 2016) or cheese made from it (Hunt et al., 2009). A detailed understanding of the prevalence and characteristics of *C. jejuni* on dairy cattle farms, which are likely to become sources of contamination (Young et al., 2007), is required.

The *C. jejuni* farm-level prevalence measured in our study was 60.0%, which is higher than that detected in other temperate countries, such as Canada (6.5%) (Guevremont et al., 2014), but similar to that on English and Welsh farms (62.5%) (Ellis-Iversen et al., 2009). Differences in prevalence among various studies may result from differences in study size, sampling design, region, and seasonality (Stanley et al., 1998). Nevertheless, because *C. jejuni* on farms can be introduced to humans through various means, such as through contaminated milk or direct contact (Oliver et al., 2005), the high prevalence (60.0%) of *C. jejuni* on cattle farms may pose a potential risk to the public.

The results of multivariable GEE analysis indicated that a high density of cattle is more likely to be associated with a higher prevalence of *C. jejuni* on farms. This result suggests that reducing the animal density in the herd unit may result in a reduction in the occurrence of *C. jejuni* on farms. Social stresses such as social isolation, instability, and crowding can change pathogen exposure and transmission dynamics among individuals (Proudfoot and Habing, 2015). They can also lead to changes in the immunity of individuals and increase pathogen shedding (Aich et al., 2007; Wilcox et al., 2013).

Based on our analysis, farms with an average environmental temperature below 24°C over the 7 days prior to sampling were more likely to be associated with higher prevalence of *C. jejuni*, compared with farms with the average temperature above 24°C. A meta-analysis of the association between temperature and the survival time of *Campylobacter* also supports this: Membre et al. (2013) have shown that while *C. jejuni* does not grow at low temperatures, it survives for longer periods than at higher temperatures. This indicates that the increased survival of *C. jejuni* in farm environments with temperatures below 24°C could increase the likelihood of re-infection in other cattle in the same herd through fecal to oral transmission.

Among cattle-derived isolates specifically, 12 different profile types were identified, and virulence profile type 02 (profile type 01 + *hcp*) was predominant. Seven profile types harboring the *hcp* gene were identified among the 12 types observed among isolates from cattle sources. Based on a comparison of the prevalence of virulence-associated genes by source, the *hcp* (hemolysin co-regulated protein) gene was detected at a higher frequency among *C. jejuni* isolates from dairy cattle (58.3%) than among *C. jejuni* isolates from chickens (42.3%) or humans (27.5%). *hcp*-positive *C. jejuni* is known to be associated with bloody diarrhea in humans (Harrison et al., 2014). Similarly, our results suggest that *C. jejuni* isolated from dairy cattle carry a high potential risk when they are transmitted to humans. To our knowledge, this is the first study to compare the prevalence of the *hcp* gene in *C. jejuni* isolated from cattle with its prevalence among other sources (chickens and humans).

In many molecular epidemiological studies for human campylobacteriosis, most of the *C. jejuni* strains belonged to CC-21, but the proportions differed according to sampling time and region, ranging from 17.6 to 64.0% in several countries. CC-21 *C. jejuni* with “multihost” characteristics have been isolated from various agricultural and environmental sources (Colles and Maiden, 2012). CC-45 is the second most common CC in many studies, but in some cases, CC-42, CC-48, or CC-206 were the second most common CC (Manning et al., 2003; Nielsen et al., 2010; Mughini Gras et al., 2012; Thepault et al., 2018). Compared to *C. jejuni* strains from cattle sources from other studies, the frequencies of CC-21 and CC-48 were relatively high, and unlike other studies, CC-61 was detected specifically (Manning et al., 2003; Kwan et al., 2008; Thepault et al., 2018). However, the low proportion of CC-21 among chicken strains was similar to that observed in previous studies, in which the prevalence of CC-21 ranged from 0 to 25.14% for *C. jejuni* isolated from chicken sources from several countries (Guyard-Nicodeme et al., 2015; Llarena et al., 2015; Prachantasena et al., 2016; Ramonaite et al., 2017). Considering the results of previous studies together with our findings, the MLST distributions of *C. jejuni* isolates from three different sources showed the possibility of clonal relationships between isolates from human and cattle sources, mainly clustered in CC-21 and CC-48 according to MST analysis (Figures 2, 3).

Considering the range of hosts and clonality of CC-21 and CC-48 in our MLST results, genotyping methods with greater resolution (composite analysis of PFGE and *flaA*-RFLP typing) than that of MLST are needed to differentiate clonal strains of *C. jejuni* from multiple sources for tracking the primary origin. Based on composite analysis of PFGE and *flaA*-RFLP data from the two representative CCs exhibiting clonality, we found that cattle strains exhibited patterns that were indistinguishable from human strains within CC-21 (Figure 4A, cluster 5), indicating that *C. jejuni* from dairy cattle may be transmitted to humans through complex routes. In the case of CC-48, it was observed that *C. jejuni* isolates from cattle sources were clustered together with chicken and human strains (Figure 4B, cluster 1), indicating that strains from diverse sources may be transmitted through complex routes between livestock, the environment, and humans. Generally, infections are caused by the ingestion of contaminated livestock products such as raw milk or meat (Young et al., 2007). In addition, transmission patterns between the environment, farm animals, wild animals, and humans interact in complex ways with various ecological factors (Bronowski et al., 2014). For example, *C. jejuni*, which is present in the feces of livestock, can contaminate water and may also be directly transmitted to humans through the aquatic environment (Young et al., 2007).

We found that higher proportions of *hcp* and *virB11*, which are related to *C. jejuni* secretion systems and are involved in pathogenicity, in isolates from dairy cattle sources compared to those from other sources (Table 3). In addition, higher levels of *hcp* and *virB11* were detected in *C. jejuni* from cattle belonging to CC-21 and CC-48, which clustered with human strains. Since the pVir plasmid containing the *virB11* gene plays an important role in the invasion of the intestinal epithelial cells, this suggests that infection with *C. jejuni* carrying *virB11* may lead to more severe clinical symptoms (Tracz et al., 2005). High *C. jejuni* prevalence on dairy cattle farms and higher proportion of *hcp* and *virB11* genes of *C. jejuni* from cattle source compared to other sources indicated that the *C. jejuni* isolated from dairy cattle pose a potential risk to humans when *hcp*- and/or *virB11*-positive *C. jejuni* were transmitted. In fact, 34.4% of the foodborne *Campylobacter* outbreaks in the United States from 2010 to 2015 were attributed to dairy products (Centers for Disease Control and Prevention, 2017). However, it can be difficult to prevent such outbreaks and take effective precautions in advance since the detection of *Campylobacter* in bulk milk is challenging (Bianchini et al., 2014), and the importance of improving the detection of *Campylobacter* in dairy products (milk samples) has been emphasized recently (Razzuoli et al., 2018). Therefore, our investigation into the virulence profiles and genetic relationships among *Campylobacter* strains isolated from human clinical cases and *C. jejuni* strains isolated from dairy cattle (primary origin) on farms and the results of our association analysis of the farm environment may help to control *Campylobacter* prevalence at the farm-level and reduce its spread through the environment or dairy products.

In conclusion, the MLST distributions of *C. jejuni* isolates from three different sources showed clonal relationships between isolates from human and cattle sources, mainly clustered in CC-21 and CC-48. In addition, we found that cattle isolates shared indistinguishable pattern with human isolates within these CCs based on composite analysis of PFGE and *flaA*-RFLP typing. We suggest that high prevalence of *C. jejuni* on cattle farms and high virulence-associated gene (*hcp* and *virB11*) detection rates not only provide a decisive clue to a close genetic relationship between cattle- and human-derived strains, but also demonstrate the high potential risks to humans associated with dairy products or the dairy farm environment. Considering the GEE analysis of risk factors associated with *C. jejuni* prevalence on dairy cattle farms, we suggest that the presence and survival of *C. jejuni* in the farm environment could play an important role in re-infecting cattle on the same farm, making dairy cattle a potential reservoir of human campylobacteriosis. Therefore, improvements to the environmental management of cattle farms could reduce the shedding of *C. jejuni* from cattle, thereby reducing the potential risk of *C. jejuni* at the farm-level and its spread to humans through the food chain.

## Author Contributions

SC conceived and designed the study. HH and W-HK analyzed the epidemiologic data. HH, JoK, W-HK, JuK, and SH performed sampling. SL, S-HM, and J-HG prepared the manuscript. J-UA was a major contributor, both in experiments and writing the manuscript. All authors have read and approved the final manuscript.

## Conflict of Interest Statement

The authors declare that the research was conducted in the absence of any commercial or financial relationships that could be construed as a potential conflict of interest.
