# Supplementary material for: Dairy Cattle, a Potential Reservoir of Human Campylobacteriosis: Epidemiological and Molecular Characterization of Campylobacter jejuni From Cattle Farms
Source: Front Microbiol. 2018 Dec 18;9:3136. doi: 10.3389/fmicb.2018.03136 (PMC6305296; doi:10.3389/fmicb.2018.03136)
Supplement: Supplementary file 1 [file Table_1.DOCX]

Supplementary Material

Dairy cattle, a potential reservoir of human campylobacteriosis: epidemiological and molecular characterization of *Campylobacter jejuni* from cattle farms

**Jae-Uk An^1^, Hungwui Ho^2^, Jonghyun Kim^3^, Woo-Hyun Kim^1^, Junhyung Kim^1^, Soomin Lee^1^, Seung-Hyun Mun^1^, Jae-Ho Guk^1^, Sahyun Hong^4^, Seongbeom Cho^1,*^**

^1^BK21 PLUS Program for Creative Veterinary Science Research, Research Institute for Veterinary Science and College of Veterinary Medicine, Seoul National University, Seoul, Republic of Korea

^2^Veterinary Research Institute, 59, Jalan Sultan Azlan Shah, 31400, Ipoh, Perak, Malaysia

^3^Division of Bacterial Disease Research, Center for Infectious Diseases Research, Korea National Institute of Health, Centers for Disease Control and Prevention, Cheongju-si, Chungcheongbuk-do, 28159, Republic of Korea

^4^Division of Bacterial Disease, Center for Laboratory Control of Infectious Diseases, Centers for Disease Control and Prevention, Cheongju-si, Chungcheongbuk-do, 28159, Republic of Korea

*** Correspondence:** Seongbeom Cho: [chose@snu.ac.kr](mailto:chose@snu.ac.kr)

# Supplementary Figures and Tables

## Supplementary Figures

**
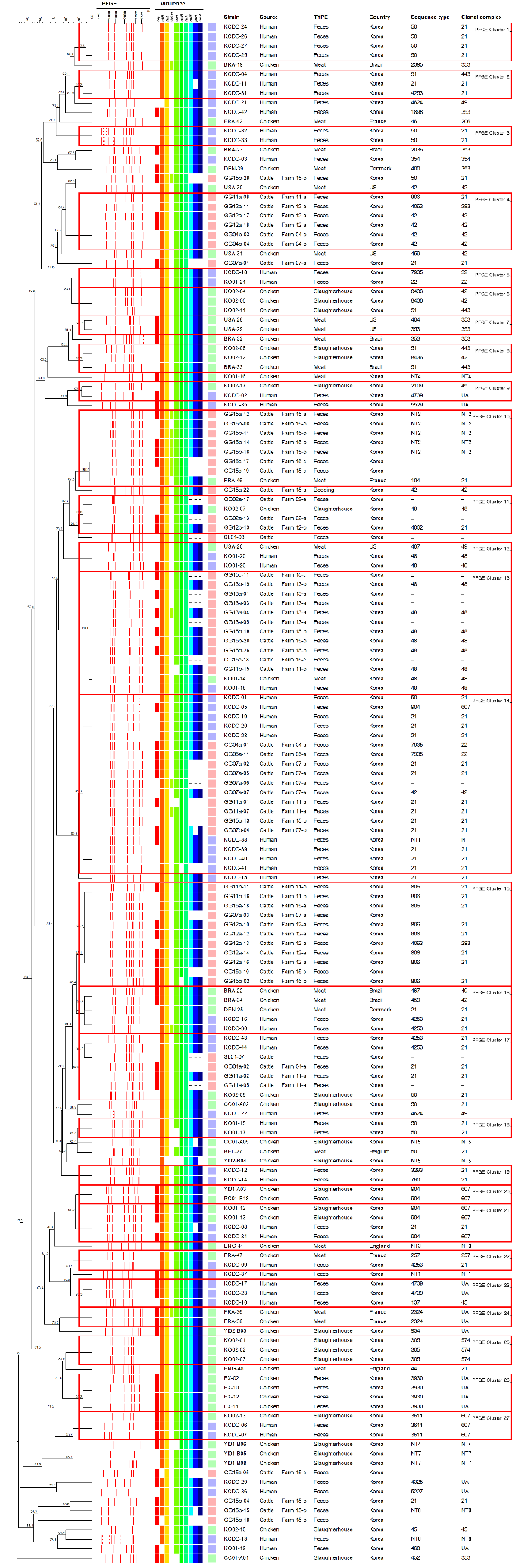
**

**Supplementary Figure 1.** **UPGMA dendrogram based on PFGE band pattern analysis of 163 *C. jejuni* strains from all three sources.** Dendrogram was generated based on 90% similarity and a 1.5% tolerance for PFGE. The letter next to the farm number indicates the visit number (a-first visit, b-second visit, c-third visit, d-fourth visit). Hyphens for virulence-associated genes and MLST results indicate missing data.

**
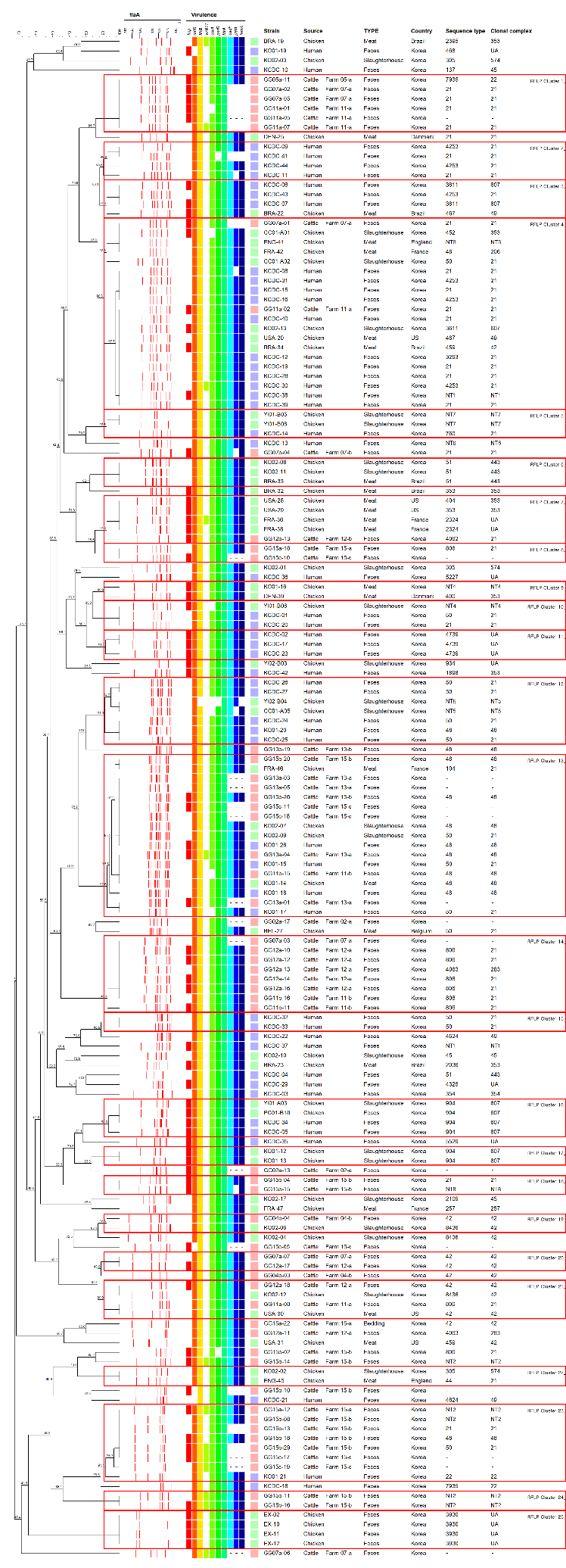
**

**Supplementary Figure 2.** **UPGMA dendrogram based on *flaA*-RFLP band pattern analysis of 163 *C. jejuni* strains from all three sources.** Dendrogram was generated based on 90% similarity and a 1.5% tolerance for *flaA-*RFLP typing. The letter next to the farm number indicates the visit number (a-first visit, b-second visit, c-third visit, d-fourth visit). Hyphens for virulence-associated genes and MLST results indicate missing data.

**
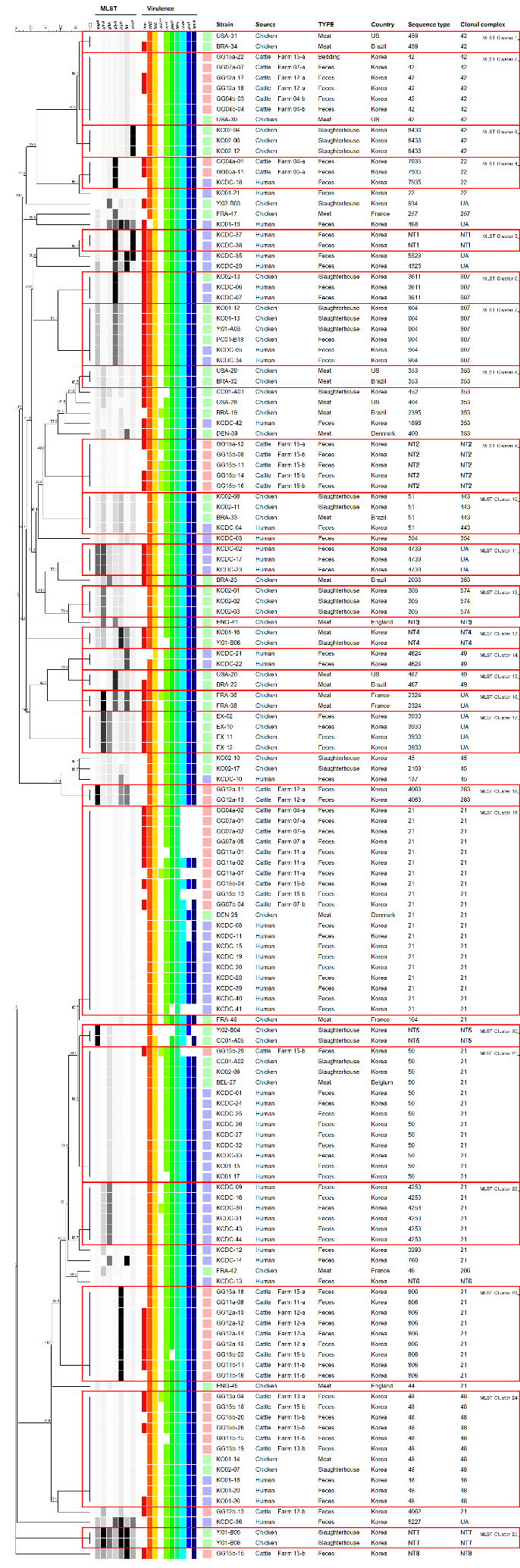
**

**Supplementary Figure 3.** **UPGMA dendrogram based on MLST data of 146 *C. jejuni* strains from all three sources.** Dendrogram was generated using cluster analysis based on categorical coefficients. The letter next to the farm number indicates the visit number (a-first visit, b-second visit, c-third visit, d-fourth visit). Data from 17 isolates were missing during study.

## Supplementary Tables

**Supplementary Table 1.** Epidemiological characteristics of 15 cattle farms included in this study and their *C. jejuni* prevalences.

| Farm | Farm size | Housing density of herd^a^ | Sawdust hygiene level^b^ | Stamping out  due to FMD | Main feed | Total of visit | Year | *C. jejuni* positive isolates/ samples collected (%) |
| --- | --- | --- | --- | --- | --- | --- | --- | --- |
| 1 | 250 | Normal | High | No | TMR^c^ | 1 | 2012 | 0/8 ( 0.0) |
| 2 | 60 | Normal | Moderate | Yes | TMR | 1 | 2012 | 2/10 (20.0) |
| 3 | 60 | High | Low | No | TMR | 1 | 2012 | 2/8 (25.0) |
| 4 | 50 | High | Low | No | Hay | 2 | 2012 | 2/9 (22.2) |
|  |  |  |  |  |  |  | 2013 | 2/6 (33.3) |
| 5 | 30 | High | Low | No | TMR | 1 | 2012 | 1/7 (14.3) |
| 6 | 40 | Normal | High | No | TMR | 1 | 2012 | 0/9 ( 0.0) |
| 7 | 160 | Normal | High | Yes | TMR | 2 | 2012 | 7/10 (70.0) |
|  |  |  |  |  |  |  | 2013 | 1/8 (12.5) |
| 8 | 150 | Normal | High | Yes | TMR | 1 | 2012 | 0/10 ( 0.0) |
| 9 | 50 | Normal | High | No | TMR | 1 | 2012 | 0/10 ( 0.0) |
| 10 | 70 | Normal | Low | No | TMR | 2 | 2012 | 0/10 ( 0.0) |
|  |  |  |  |  |  |  | 2013 | 0/4 ( 0.0) |
| 11 | 30 | Normal | Moderate | Yes | TMR | 2 | 2012 | 6/9 (66.7) |
|  |  |  |  |  |  |  | 2013 | 5/7 (71.4) |
| 12 | 50 | Normal | Low | Yes | TMR | 2 | 2012 | 8/10 (80.0) |
|  |  |  |  |  |  |  | 2013 | 2/9 (22.2) |
| 13 | 80 | Normal | High | No | TMR | 2 | 2012 | 7/11 (63.6) |
|  |  |  |  |  |  |  | 2013 | 3/8 (37.5) |
| 14 | 60 | Normal | High | Yes | TMR | 1 | 2012 | 0/12 ( 0.0) |
| 15 | 150 | Normal | High | No | Hay | 4 | 2012 | 3/24 (12.5) |
|  |  |  |  |  |  |  | 2012 | 0/28 ( 0.0) |
|  |  |  |  |  |  |  | 2013 | 14/32 (43.6) |
|  |  |  |  |  |  |  | 2013 | 6/36 (16.7) |
| Total |  |  |  |  |  | 24 |  | 71/295 (24.0) |

^a^Housing density of animals in the lactating herd: Normal, ≥8.3 m²/cattle; High, <8.3 m²/cattle (Ministry of Government legislation, 2017).

^b^Hygiene level of sawdust: Low; very wet, dirty, and slippery, Moderate; normal, High; very dry (Reneau et al., 2005).

^c^TMR: Total mixed ration.

**Supplementary Table 2.** Oligonucleotide primers and PCR conditions used in this study.

| Gene | Primer | Nucleotide sequences (5’ to 3’) | Amplicon  size (bp) | PCR conditions | References |
| --- | --- | --- | --- | --- | --- |
| 16S rRNA | F | GGATGACACTTTTCGGAGC | 816 | 95°C for 15 min/ 25 cycles of 95°C x 30 s, 58°C x 1 min 30 s and 72°C x 1 min/ 72°C for 7 min^a^ | (Yamazaki-Matsune et al., 2007) |
|  | R | CATTGTAGCACGTGTGTC |  |  |  |
| *cj0414* | F | CAAATAAAGTTAGAGGTAGAATGT | 161 |  |  |
|  | R | CCATAAGCACTAGCTAGCTGAT |  |  |  |
| *hipO* | F | GCAAAATCCACAGCTTCATCG | 350 |  | (Unpublished data) |
|  | R | GGAAGGGGTGGTCATGGAAG |  |  |  |
| *flaA* | F | GGATTTCGTATTAACA | 1728 | 94°C for 10 min/ 35 cycles of 94°C x 1 min, 45°C x 1 min and 72°C x 2 min/ 72°C for 7 min | (El-Adawy et al., 2013; Nachamkin et al., 1996) |
|  | R | CTGTAGTAATCTTAAAACATTTTG |  |  |  |
|  | R-Wob^b^ | CTGTARYAATCTTAAAACATTTTG |  |  | (El-Adawy et al., 2013) |
| *flhB* | F | TGGCAGGCGAAGATCAAGAA | 549 | 95°C for 1 min/ 35 cycles of 95°C x 1 min, 55°C x 1 min and 72°C x 1 min/ 72°C for 5 min | (Koolman et al., 2015) |
|  | R | GCCAAGTAAGCTGTGCAACC |  |  |  |
| *virB11* | F | TCAGGTGGAACAGGAAGTGG | 329 | 95°C for 1 min/ 35 cycles of 95°C x 1 min, 54°C x 1 min and 72°C x 1 min/ 72°C for 5 min | (Koolman et al., 2015) |
|  | R | GCTTTGATCGCGTCTTCTGG |  |  |  |
| *hcp* | F | CAAGCGGTGCATCTACTGAA | 463 | 95°C for 2 min/ 30 cycles of 95°C x 15 s, 54°C x 30 s and 72°C x 30 s / 72°C for 5 min | (Harrison et al., 2014) |
|  | R | TAAGCTTTGCCCTCTCTCCA |  |  |  |
| *cdtB* | F | GCTCCTACATCAACGCGAGA | 376 | 95°C for 1 min/ 35 cycles of 95°C x 1 min, 55°C x 1 min and 72°C x 1 min/ 72°C for 5 min | (Koolman et al., 2015) |
|  | R | ACTACTCCGCCTTTTACCGC |  |  |  |
| *perR* | F | CCCTTCAATCTCTTTAGCGACG | 153 | 95°C for 4 min/ 30 cycles of 95°C x 30 s, 55°C x 30 s and 72°C x 30 s / 72°C for 7 min | This study |
|  | R | ATACCACCACATTTGGCGCA |  |  |  |
| *csrA* | F | CACAGTCAGTGAAGGTGCTT | 878 | 94°C for 3 min/ 30 cycles of 94°C x 30 s, 58°C x 30 s and 72°C x 30 s/ 72°C for 5 min | (González-Hein et al., 2013) |
|  | R | ACTCGCACAATCGCTACTTC |  |  |  |
| *htrA* | F | CCATTGCGATATACCCAAACTT | 130 | 94°C for 5 min/ 30 cycles of 94°C x 15 s, 52°C x 20 s and 72°C x 15 s/ 72°C for 3 min | (Bui et al., 2012) |
|  | R | CTGGTTTCCAAGAGGGTGAT |  |  |  |
| *cadF* | F | TTGAAGGTAATTTAGATATG | 400 | 94°C for 1 min/ 30 cycles of 94°C x 30 s, 48°C x 1 min and 72°C x 1 min/ 72°C for 5 min | (Konkel et al., 1999) |
|  | R | CTAATACCTAAAGTTGAAAC |  |  |  |
| *pldA* | F | AAGCTTATGCGTTTTT | 913 | 94°C for 1 min/ 30 cycles of 94°C x 30 s, 48°C x 1 min and 72°C x 1 min/ 72°C for 5 min | (Datta et al., 2003) |
|  | R | TATAAGGCTTTCTCCA |  |  |  |
| *iamA* | F | GCACAAAATATATCATTACAA | 518 | 94°C for 1 min/ 30 cycles of 94°C x 30 s, 55°C x 1 min and 72°C x 1 min/ 72°C for 5 min | (Muller et al., 2006) |
|  | R | TTCACGACTACTATGAGG |  |  |  |

^a^Multiplex PCR conditions for the 16S rRNA gene and *cj0414*.

^b^*flaA*-R-Wob primer was used as the reverse primer if the first set of primers (*flaA*-F and *flaA*-R) did not amplify *flaA*.

**Supplementary Table 3.** Diversity indices of subtyping methods used for *C. jejuni* isolates from different sources.

|  |  | Number of  isolates | Number of  genotypes | Simpson’s diversity index | | Shannon's diversity index^c^ | |
| --- | --- | --- | --- | --- | --- | --- | --- |
| Sources |  |  |  | 1-D | 95% Cl | H' | *P* value |
| All sources^a^ | PFGE | 163 | 66 | 0.967 | 0.956- 0.979 | 3.729 | - |
|  | *flaA-*RFLP | 159^b^ | 63 | 0.962 | 0.948- 0.975 | 3.640 | 0.397 |
|  | MLST | 146 | 51 | 0.957 | 0.942- 0.971 | 3.449 | 0.008 |
| Cattle farm | PFGE | 60 | 15 | 0.887 | 0.856- 0.918 | 2.278 | 0.000 |
|  | *flaA*-RFLP | 56^b^ | 25 | 0.933 | 0.903- 0.963 | 2.821 | - |
|  | MLST | 43^c^ | 10 | 0.864 | 0.823- 0.905 | 2.014 | 0.000 |
| Chicken | PFGE | 52 | 30 | 0.962 | 0.937- 0.988 | 3.165 | 0.126 |
|  | *flaA*-RFLP | 52 | 30 | 0.973 | 0.961- 0.985 | 3.255 | 0.388 |
|  | MLST | 52 | 30 | 0.976 | 0.965- 0.987 | 3.271 | - |
| Human | PFGE | 51 | 25 | 0.947 | 0.911- 0.985 | 2.960 | - |
|  | *flaA*-RFLP | 51 | 25 | 0.889 | 0.818- 0.961 | 2.676 | 0.099 |
|  | MLST | 51 | 22 | 0.924 | 0.886- 0.961 | 2.708 | 0.071 |

^a^Isolates from all sources: cattle, chickens, and humans.

^b^Four isolates (two from cattle slaughterhouses, two from farm 4) were untypeable by *flaA-*RFLP typing.

^c^Data from 17 isolates were missing during study
